# Supplementary material for: Spin-chirality-driven second-harmonic generation in two-dimensional magnet CrSBr
Source: Sci Adv. 2025 Apr 4;11(14):eadu6562. doi: 10.1126/sciadv.adu6562 (PMC11970475; doi:10.1126/sciadv.adu6562)
Supplement: Supplementary file 1 — Sections S1 to S3 Figs. S1 to S19 [file sciadv.adu6562_sm.pdf]

Science Advances
AAAS

Supplementary Materials for

Spin-chirality–driven second-harmonic generation in two-dimensional magnet CrSBr

Dezhao Wu *et al.*

Corresponding author: Yong Xu, yongxu@mail.tsinghua.edu.cn; Meng Ye, mye@gscaep.ac.cn

Sci. Adv. **11**, eadu6562 (2025)
DOI: 10.1126/sciadv.adu6562

This PDF file includes:

Sections S1 to S3
Figs. S1 to S19

1 SHG of 2D CrSBr

1.1 Influence of interlayer coupling

In odd-layer CrSBr, the $\mathcal{P}$ symmetry is restored, leading to the vanishing of ED-SHG. In even-layer CrSBr, both the c-type MSHG susceptibility $|\chi^{xxx}|$ and the chiral SHG susceptibility $|\chi^{yyy}|$ are found to be insensitive to layer number, as shown in Fig. S1B. As shown in Fig. S1A, the layer pair consisting of neighboring bilayer (BL) cAFM CrSBr in the green rectangle is labeled as layer pair 1. It is related to the adjacent layer pair in the purple rectangle labeled layer pair 2 by the $\mathcal{P}$ operation. Assuming that each layer pair induces an SHG signal χ, the $\mathcal{P}$ operation will reverse the sign of the SHG and induce an opposite signal $-\chi$. Consequently, in even-layer structures such as 4L cAFM CrSBr, the total SHG signal is equal to that of a BL structure. This layer-independent behavior of SHG in 2D CrSBr is in good agreement with the experimental results (*3*).

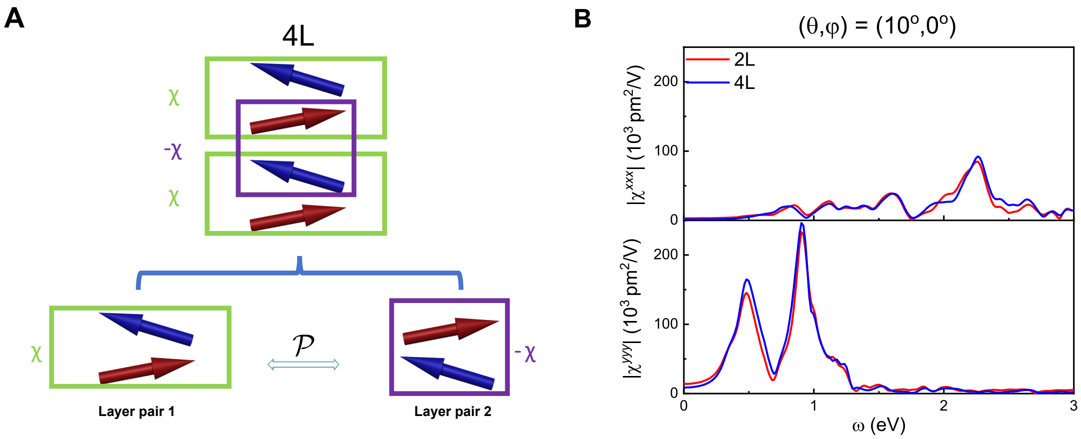

Figure S1: **Influence of layer thickness on SHG in 2D cAFM CrSBr at (10°,0°) canting state.** (A) Schematics of the 4L of cAFM CrSBr. (B) The c-type MSHG susceptibility $|\chi^{xxx}|$ and the chiral SHG susceptibility $|\chi^{yyy}|$ in cAFM CrSBr with different layer numbers.

Although BL and 4L cAFM CrSBr show similar MSHG in Fig. S1B, we proposed that BL CrSBr is optimal for observing chiral SHG or c-type MSHG. The reasons are multiple.

In our study, the results are presented in terms of the two-dimensional (2D) SHG susceptibility χ_{2D}, which is suitable for describing 2D materials, as it denotes the intensity of SHG per unit area (in units of 10^3 pm^2/V). Distinctly, bulk materials are described by the three-dimensional (3D) susceptibility χ_{3D}, which refers to the intensity of the SHG per unit volume (in units of pm/V). The two types of susceptibility are related by

$$\chi_{2D} = \chi_{3D} \times Z_{\text{eff}} \,, \tag{S1}$$

where Z_{eff} is the effective thickness of the 2D material. As one can see, directly comparing the SHG intensity between 2D and bulk materials is somewhat tricky, as χ_{2D} and χ_{3D} have

different units. Figure. S1B actually demonstrates that both BL and 4L exhibit similar χ_{2D} values, indicating that the SHG susceptibility per unit area remains comparable across these thicknesses. However, to compare with bulk CrSBr, we need to convert χ_{2D} to χ_{3D}. As shown in Fig. S2, there is a clear decay of χ_{3D} with an increase in the number of CrSBr layers, and χ_{3D} is much smaller in bulk. If experimental SHG measurements are interpreted in terms of a fixed volume within the illuminated region, as shown in Fig. S2, the SHG signal would decrease with increasing layer thickness.

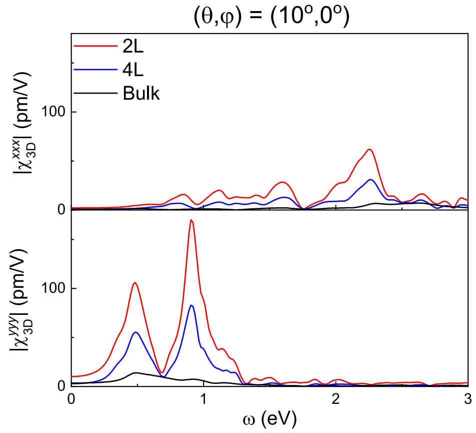

Figure S2: **3D SHG susceptibility.** 3D SHG susceptibility of c-type $|\chi_{3D}^{xxx}|$ and chiral $|\chi_{3D}^{yyy}|$ in cAFM CrSBr at the (10°, 0°) canting state for different layer numbers.

Even if the experimental observations are interpreted in terms of χ_{2D}, which reflects the SHG per unit of area, additional factors can cause a reduction in the observed SHG signal as the number of layers increases.

One is the phase-matching problem. When considering phase matching, the measured SHG intensity becomes (*43*)

$$\begin{aligned} I_{2\omega}(Z_{\text{eff}}) &= \frac{2[\chi_{3D}]^2\omega_3^2 I_\omega^2}{n_\omega^2 n_{2\omega}\epsilon_0 c^3} Z_{\text{eff}}^2 \operatorname{sinc}^2\left(\frac{\Delta k Z_{\text{eff}}}{2}\right) \\ &= \frac{2[\chi_{2D}]^2\omega_3^2 I_\omega^2}{n_\omega^2 n_{2\omega}\epsilon_0 c^3} \operatorname{sinc}^2\left(\frac{\Delta k Z_{\text{eff}}}{2}\right). \end{aligned} \tag{S2}$$

The Δk is the wave vector mismatch between the fundamental and second harmonic waves, defined as $\Delta k = 2k_\omega - k_{2\omega}$, and $\operatorname{sinc}(x) = \sin x/x$ which decays rapidly away from $x = 0$. Consequently, the intensity is strongly determined by the phase mismatch $\Delta k Z_{\text{eff}}$ and the 2D SHG coefficient χ_{2D}. As χ_{2D} remains almost the same with increasing layer number (as in the case of 2D cAFM CrSBr), the value of the measured SHG intensity is maximized when $\Delta k Z_{\text{eff}}$ is minimized, which can be achieved by $Z_{\text{eff}} \to 0$. This suggests that thinner samples of CrSBr would produce the strongest SHG signal. Generally, the phase-matching problem becomes more

notable in thicker samples, leading to a decrease in the measurable SHG intensity. Therefore, to maximize the MSHG signal, a minimal number of layers is preferred.

Another problem is the influence of magnetic polymorph states. As the number of layers increases, CrSBr may exhibit various magnetic polymorph states due to the influence of external fields and differences in sample quality, as proposed in very recent experiments (*44*). For example, as shown in Fig. S3A, 4L CrSBr can display not only the usual state with A-type AFM coupling but also a mixed state where FM and AFM couplings coexist between layers, which we denoted as 4L-MIX. The MSHG signal contributed by different layer pairs in 4L-MIX CrSBr can be roughly estimated to be zero, as depicted in Fig. S3A. Taking the $(10°, 0°)$ canting state as an example, the calculation results indeed show that both chiral SHG and c-type MSHG in 4L-MIX CrSBr are smaller than those in 4L-AFM CrSBr, as shown in S3B.

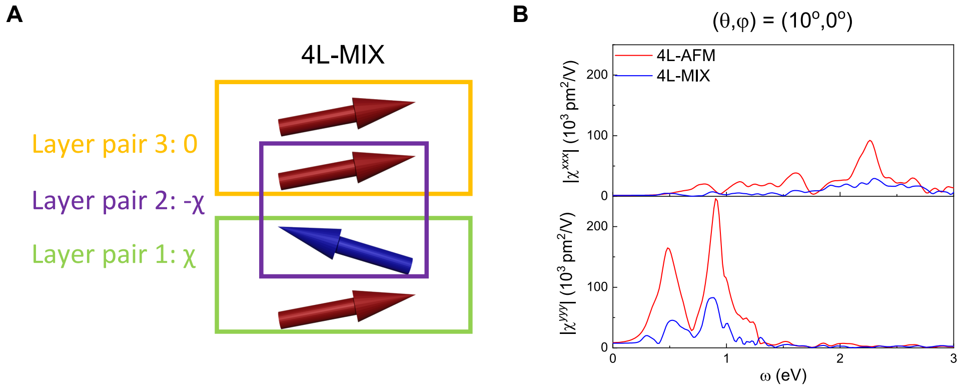

Figure S3: **Influence of magnetic polymorph states on MSHG in 2D cAFM CrSBr at the (10°,0°) canting state.** (A) Schematic of the 4L-MIX cAFM CrSBr. (B) The c-type MSHG susceptibility $|\chi^{xxx}|$ and the chiral SHG susceptibility $|\chi^{yyy}|$ in 4L cAFM CrSBr for different magnetic polymorph states.

In addition to the number of layers, the interlayer distance d can notably affect the SHG in BL CrSBr. The spectral integrals intuitively reveal that both c-type MSHG susceptibility $|\chi^{xxx}|$ and chiral SHG susceptibility $|\chi^{yyy}|$ are negatively dependent on the interlayer distance, as shown in Fig. S4, indicating the important role of interlayer coupling in generating these SHG signals.

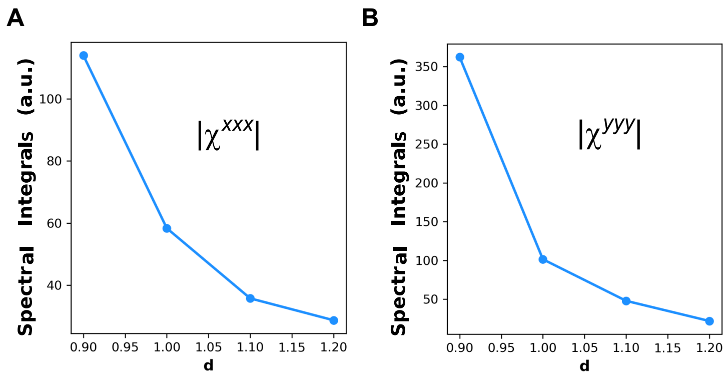

Figure S4: **Dependence of SHG spectral integrals on interlayer distance at the (10°,0°) canting state.** The spectral integrals of both the *c*-type MSHG susceptibility $|\chi^{xxx}|$ (A) and the chiral SHG susceptibility $|\chi^{yyy}|$ (B) show a negative dependence on the interlayer distance. The interlayer distance d is in units of d_0 which is the interlayer distance in the equilibrium state of BL CrSBr.

1.2 Influence of scissors correction and band gap

In our study, we employed the most widely used Perdew-Burke-Ernzerhof (PBE) exchange-correlation functional within the density functional theory (DFT), which has been extensively used to explore the electronic properties of CrSBr. It is well known that PBE, like many other exchange-correlation functionals within the standard DFT framework, systematically underestimates band gaps due to limitations in accurately describing the exchange-correlation energy and the absence of quasiparticle effects. Scissors correction is a commonly used method to roughly estimate the influence of the underestimated band gap on optical properties (*60*). This method involves rigidly shifting the conduction bands to match the experimental band gap, thereby preserving the qualitative features of the band structure while achieving quantitative accuracy. Since the band gap of CrSBr observed in previous experiments is 1.5 eV (*19*), we applied the scissors correction to shift the band gap to 1.5 eV in the SHG calculations to demonstrate its influence. As shown in Fig. S5, taking the (10°, 0°) canting state as an example, both *c*-type MSHG and chiral SHG components decrease compared to those under the original PBE band gap (about 0.6 eV). Additionally, locations of the SHG peaks shift to higher-energy regions. However, the chiral SHG components still possess considerable values. The anisotropy of the chiral SHG persists, and the maximum of chiral SHG susceptibility $|\chi^{yyy}|$ is still larger than that of any intrinsic *c*-type MSHG component, even at such a small canting angle. The above results demonstrate the robustness of our findings with respect to the scissors correction. Moreover, notable interference effects also persist, as shown in Figs. S5 (B and C). This is because the main results of our work—including the emergence of chiral SHG at the expected tensor components and its proportionality to spin chirality, as well as the interference effects are ensured by symmetry and thus unaffected by the band gap.

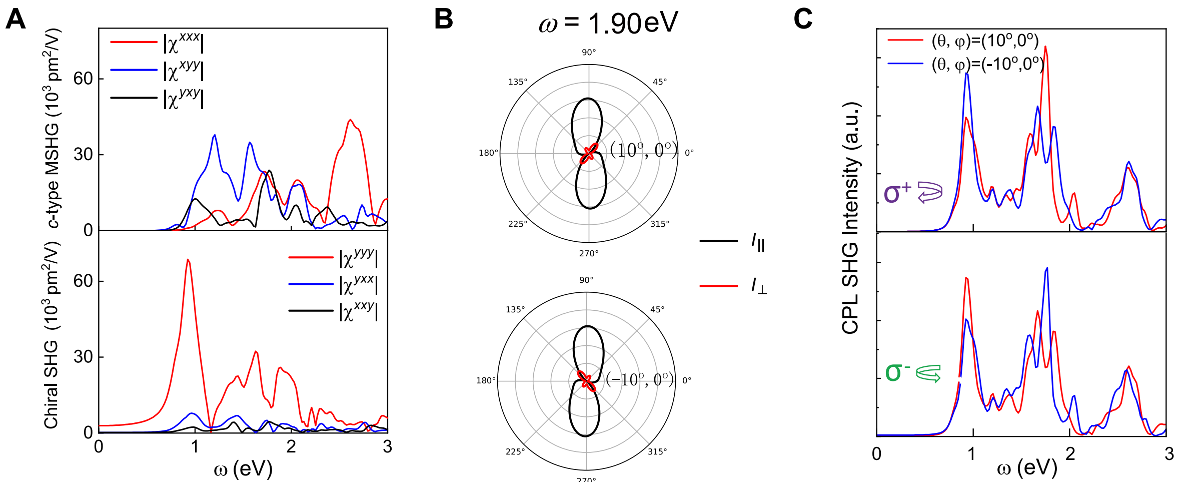

Figure S5: **SHG under a band gap of 1.5 eV at the (10°,0°) canting state.** (A) Norm of all in-plane SHG susceptibilities at the $(10°, 0°)$ canting state. The upper panel displays the c-type MSHG components, while the lower panel shows the chiral SHG components. The polarization-resolved SHG at ω =1.9 eV (B) and circularly polarized SHG intensity (C) under $(\pm10°, 0°)$ canting states.

1.3 Influence of quantum decoherence

The broadening factor η applied to the Dirac delta function in calculations of SHG susceptibility (see formulas in Section S3.2) effectively reflects the influence of quantum decoherence and it is related to the phenomenological decoherence time τ (see Eq. A7 in our previous work (*29*)) via the relation

$$\eta = \frac{\hbar}{\tau}. \tag{S3}$$

The broadening factor $\eta = 0.05$ eV in our calculations corresponds to $\tau = 13$ fs, which has been widely used in the calculations of SHG (*4*, *29*). To further investigate the influence of quantum decoherence, we performed additional calculations using different η v alues. Based on previous theoretical works (*57*, *58*), several to tens of femtoseconds are considered typical decoherence times for 2D materials, which is also supported by experiments (*59*). We therefore used additional $\eta = 0.033$ eV and 0.132 eV, corresponding to the decoherence times $\tau = 20$ fs and 5 fs, respectively. The corresponding SHG results, along with the previous calculation results under $\tau = 13$ fs are shown in Fig. S6. One can find that extending the quantum coherence by introducing longer τ leads to an enhancement of both chiral SHG and c-type MSHG signals, with the chiral SHG being more sensitive to this enhancement. This indicates that large chiral SHG, as well as giant interference effects arising from the comparable magnitudes of chiral SHG and c-type SHG, can be observed at smaller spin canting angles with respect to the increase in τ.

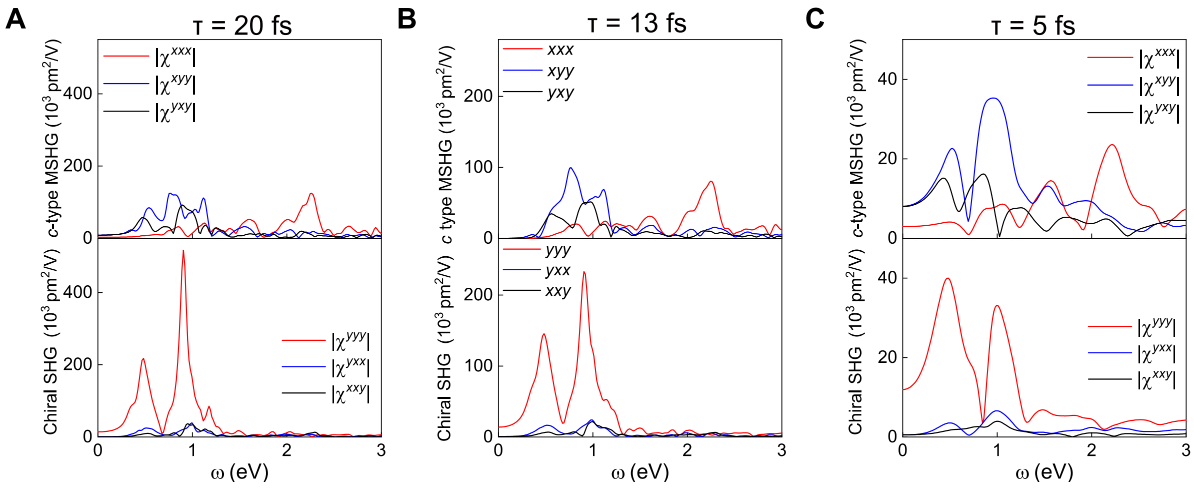

Figure S6: **The influence of decoherence time τ, which related to broadening factor η by $\eta = \hbar/\tau$, on the SHG of BL cAFM CrSBr at the (10°,0°) canting state.** SHG susceptibilities under (A) $\tau = 20$ fs ($\eta = 0.033$ eV), (B) $\tau = 13$ fs ($\eta = 0.05$ eV), and (C) $\tau = 5$ fs ($\eta = 0.132$ eV).

1.4 Supplementary materials of interference effects

The general expression of in-plane SHG polarization responses for 2D materials in normal incident geometry are given by

$$\begin{aligned} P_x(2\omega) &= \chi^{xxx} E_x^2(\omega) + 2\chi^{xxy} E_x(\omega) E_y(\omega) + \chi^{xyy} E_y^2(\omega)\,, \\ P_y(2\omega) &= \chi^{yxx} E_x^2(\omega) + 2\chi^{yxy} E_x(\omega) E_y(\omega) + \chi^{yyy} E_y^2(\omega)\,. \end{aligned} \tag{S4}$$

Under the illumination of linearly polarized light (LPL) characterized by $\mathbf{E}(\omega) = E(\omega)(\cos\phi, \sin\phi)$, they become

$$\begin{aligned} P_x(2\omega) &= (\chi^{xxx}\cos^2\phi + 2\chi^{xxy}\sin\phi\cos\phi + \chi^{xyy}\sin^2\phi) E^2(\omega)\,, \\ P_y(2\omega) &= (\chi^{yxx}\cos^2\phi + 2\chi^{yxy}\sin\phi\cos\phi + \chi^{yyy}\sin^2\phi) E^2(\omega)\,. \end{aligned} \tag{S5}$$

For BL cAFM CrSBr, under the reversal of spin-canting direction, which can be achieved by the reversal of **B** field direction, the corresponding $\mathcal{PT}$ operation reverse the sign of i-type chiral SHG components (see Table 2 in the main text) χ^{yyy}, χ^{yxx}, and χ^{xxy}. The in-plane nonlinear

polarization responses become

$$
\begin{aligned}
P'_x(2\omega) &= (\chi^{xxx}\cos^2\phi - 2\chi^{xxy}\sin\phi\cos\phi + \chi^{xyy}\sin^2\phi)E^2(\omega) \\
&= (\chi^{xxx}\cos^2\phi + 2\chi^{xxy}\sin(-\phi)\cos\phi + \chi^{xyy}\sin^2\phi)E^2(\omega) \\
&= \mathcal{M}_\phi P_x(2\omega)\,, \\
P'_y(2\omega) &= (-\chi^{yxx}\cos^2\phi + 2\chi^{yxy}\sin\phi\cos\phi - \chi^{yyy}\sin^2\phi)E^2(\omega) \\
&= -(\chi^{yxx}\cos^2\phi - 2\chi^{yxy}\sin\phi\cos\phi + \chi^{yyy}\sin^2\phi)E^2(\omega) \\
&= -(\chi^{yxx}\cos^2\phi + 2\chi^{yxy}\sin(-\phi)\cos\phi + \chi^{yyy}\sin^2\phi)E^2(\omega) \\
&= -\mathcal{M}_\phi P_y(2\omega)\,,
\end{aligned} \tag{S6}
$$

where ϕ denotes the angle between the light polarization and the $\hat{x}$-axis of the sample, and $\mathcal{M}_\phi$ represents the mirror operation of ϕ, i.e., reversing ϕ to $-\phi$. To be consistent with the experimental setup (*3*), these in-plane nonlinear polarization responses can be further decomposed into the parallel ($||$) and perpendicular ($\perp$) directions with respect to the electric field of the incident light

$$
\begin{aligned}
P_{||}(2\omega) &= P_x(2\omega)\cos\phi + P_y(2\omega)\sin\phi\,, \\
P_{\perp}(2\omega) &= -P_x(2\omega)\sin\phi + P_y(2\omega)\cos\phi\,.
\end{aligned} \tag{S7}
$$

Under $\mathcal{PT}$ operation, they become

$$
\begin{aligned}
P'_{||}(2\omega) &= P'_x(2\omega)\cos\phi + P'_y(2\omega)\sin\phi \\
&= \cos\phi\mathcal{M}_\phi P_x(2\omega) - \sin\phi\mathcal{M}_\phi P_y(2\omega) \\
&= \mathcal{M}_\phi P_x(2\omega)\cos\phi + \mathcal{M}_\phi P_y(2\omega)\sin\phi \\
&= \mathcal{M}_\phi P'_{||}(2\omega)\,, \\
P'_{\perp}(2\omega) &= -P'_x(2\omega)\sin\phi + P'_y(2\omega)\cos\phi \\
&= -\sin\phi\mathcal{M}_\phi P'_x(2\omega) - \cos\phi\mathcal{M}_\phi P'_y(2\omega) \\
&= \mathcal{M}_\phi P'_x(2\omega)\sin\phi - \mathcal{M}_\phi P'_y(2\omega)\cos\phi \\
&= -\mathcal{M}_\phi P_{\perp}(2\omega)\,.
\end{aligned} \tag{S8}
$$

It is worth noting that Eq. S8 holds for both the real and imaginary parts of nonlinear polarization responses, i.e., $\mathrm{Re}[P(2\omega)]$ and $\mathrm{Im}[P(2\omega)]$. As the SHG intensity is proportional to $|P(2\omega)|^2$,

the SHG intensity $I(2\omega)$ under the $\mathcal{PT}$ operation reads

$$
\begin{aligned}
I'_{||}(2\omega) &\propto |P'_{||}(2\omega)|^2 \\
&\propto (\mathrm{Re}P'_{||}(2\omega))^2 + (\mathrm{Im}P'_{||}(2\omega))^2 \\
&\propto (\mathcal{M}_\phi \mathrm{Re}P_{||}(2\omega))^2 + (\mathcal{M}_\phi \mathrm{Im}P_{||}(2\omega))^2 \\
&\propto \mathcal{M}_\phi \mathrm{Re}P_{||}(2\omega)\mathcal{M}_\phi \mathrm{Re}P_{||}(2\omega) + \mathcal{M}_\phi \mathrm{Im}P_{||}(2\omega)\mathcal{M}_\phi \mathrm{Im}P_{||}(2\omega) \\
&\propto \mathcal{M}_\phi (\mathrm{Re}P_{||}(2\omega))^2 + \mathcal{M}_\phi (\mathrm{Im}P_{||}(2\omega))^2 \\
&\propto \mathcal{M}_\phi I_{||}(2\omega)\,, \\
I'_{\perp}(2\omega) &\propto |P'_{\perp}(2\omega)|^2 \\
&\propto (\mathrm{Re}P'_{\perp}(2\omega))^2 + (\mathrm{Im}P'_{\perp}(2\omega))^2 \\
&\propto (-\mathcal{M}_\phi \mathrm{Re}P_{\perp}(2\omega))^2 + (-\mathcal{M}_\phi \mathrm{Im}P_{\perp}(2\omega))^2 \\
&\propto \mathcal{M}_\phi \mathrm{Re}P_{\perp}(2\omega)\mathcal{M}_\phi \mathrm{Re}P_{\perp}(2\omega) + \mathcal{M}_\phi \mathrm{Im}P_{\perp}(2\omega)\mathcal{M}_\phi \mathrm{Im}P_{\perp}(2\omega) \\
&\propto \mathcal{M}_\phi (\mathrm{Re}P_{\perp}(2\omega))^2 + \mathcal{M}_\phi (\mathrm{Im}P_{\perp}(2\omega))^2 \\
&\propto \mathcal{M}_\phi I_{\perp}(2\omega)\,.
\end{aligned}
\tag{S9}
$$

Consequently, under the $\mathcal{PT}$ operation, the polarization-resolved SHG pattern undergo a mirror operation along $\phi = 0°$. The interference effects of BL cAFM CrSBr under LPL illumination are thus reflected by the mirror operation of the polarization-resolved SHG pattern under **B** reversal.

For circularly polarized light (CPL) characterized by $\mathbf{E} = E(1, \pm i)$, where $\pm$ represents left/right-handed ($\sigma^{\pm}$) CPL, Eq. S4 still holds

$$
\begin{aligned}
P_x(2\omega) &= \chi^{xxx}E_x^2(\omega) \pm 2i\chi^{xxy}E_x(\omega)E_y(\omega) - \chi^{xyy}E_y^2(\omega) \\
&= (\chi^{xxx} \pm 2i\chi^{xxy} - \chi^{xyy})E^2(\omega)\,, \\
P_y(2\omega) &= \chi^{yxx}E_x^2(\omega) \pm 2i\chi^{yxy}E_x(\omega)E_y(\omega) - \chi^{yyy}E_y^2(\omega) \\
&= (\chi^{yxx} \pm 2i\chi^{yxy} - \chi^{yyy})E^2(\omega)\,.
\end{aligned}
\tag{S10}
$$

Eq. S10 is suitable for both real and imaginary parts of nonlinear polarization

$$
\begin{aligned}
\mathrm{Re}P_x(2\omega) &\propto \mathrm{Re}\chi^{xxx} \mp 2\mathrm{Im}\chi^{xxy} - \mathrm{Re}\chi^{xyy}\,, \\
\mathrm{Im}P_x(2\omega) &\propto \mathrm{Im}\chi^{xxx} \pm 2\mathrm{Re}\chi^{xxy} - \mathrm{Im}\chi^{xyy}\,, \\
\mathrm{Re}P_y(2\omega) &\propto \mathrm{Re}\chi^{yxx} \mp 2\mathrm{Im}\chi^{yxy} - \mathrm{Re}\chi^{yyy}\,, \\
\mathrm{Im}P_y(2\omega) &\propto \mathrm{Im}\chi^{yxx} \pm 2\mathrm{Re}\chi^{yxy} - \mathrm{Im}\chi^{yyy}\,.
\end{aligned}
\tag{S11}
$$

The corresponding SHG intensity under CPL illumination is

$$
\begin{aligned}
I(2\omega) \propto\ & |P_x(2\omega)|^2 + |P_y(2\omega)|^2 \\
\propto\ & (\mathrm{Re}\chi^{xxx} \mp 2\mathrm{Im}\chi^{xxy} - \mathrm{Re}\chi^{xyy})^2 + (\mathrm{Im}\chi^{xxx} \pm 2\mathrm{Re}\chi^{xxy} - \mathrm{Im}\chi^{xyy})^2 \\
& + (\mathrm{Re}\chi^{yxx} \mp 2\mathrm{Im}\chi^{yxy} - \mathrm{Re}\chi^{yyy})^2 + (\mathrm{Im}\chi^{yxx} \pm 2\mathrm{Re}\chi^{yxy} - \mathrm{Im}\chi^{yyy})^2\,.
\end{aligned}
\tag{S12}
$$

Again, under the $\mathcal{PT}$ operation, the sign reversal of i-type chiral SHG components χ^{yyy}, χ^{yxx}, and χ^{xxy} changes the SHG intensity

$$\begin{aligned} I'(2\omega) &\propto |P'_x(2\omega)|^2 + |P'_y(2\omega)|^2 \\ &\propto (\mathrm{Re}\chi^{xxx} \pm 2\mathrm{Im}\chi^{xxy} - \mathrm{Re}\chi^{xyy})^2 + (\mathrm{Im}\chi^{xxx} \mp 2\mathrm{Re}\chi^{xxy} - \mathrm{Im}\chi^{xyy})^2 \\ &+ (-\mathrm{Re}\chi^{yxx} \mp 2\mathrm{Im}\chi^{yxy} + \mathrm{Re}\chi^{yyy})^2 + (-\mathrm{Im}\chi^{yxx} \pm 2\mathrm{Re}\chi^{yxy} + \mathrm{Im}\chi^{yyy})^2 \\ &\propto (\mathrm{Re}\chi^{xxx} \pm 2\mathrm{Im}\chi^{xxy} - \mathrm{Re}\chi^{xyy})^2 + (\mathrm{Im}\chi^{xxx} \mp 2\mathrm{Re}\chi^{xxy} - \mathrm{Im}\chi^{xyy})^2 \\ &+ (\mathrm{Re}\chi^{yxx} \pm 2\mathrm{Im}\chi^{yxy} - \mathrm{Re}\chi^{yyy})^2 + (\mathrm{Im}\chi^{yxx} \pm 2\mathrm{Re}\chi^{yxy} - \mathrm{Im}\chi^{yyy})^2 . \end{aligned} \tag{S13}$$

It is clear that the $\mathcal{PT}$ operation reverses ± to ∓, or ∓ to ±, which demonstrates that the influence of the $\mathcal{PT}$ operation on CPL SHG intensity is equivalent to that of the reversal of CPL helicity. This is consistent with the switching of red and blue lines when comparing the upper and lower panels of Fig. 4C in the main text. From the above, it is evident that this equivalence between **B** reversal and CPL helicity reversal arises from the separated i-type and c-type SHG tensor components. Similar phenomena are also demonstrated in Ref. (*4*). The interference effects of BL cAFM CrSBr under CPL illumination are thus reflected by the SHG intensity change under the reversal of **B** or CPL helicity. It is worth noting that the SHG intensity change under the reversal of CPL helicity induces the SHG circular dichroism effect.

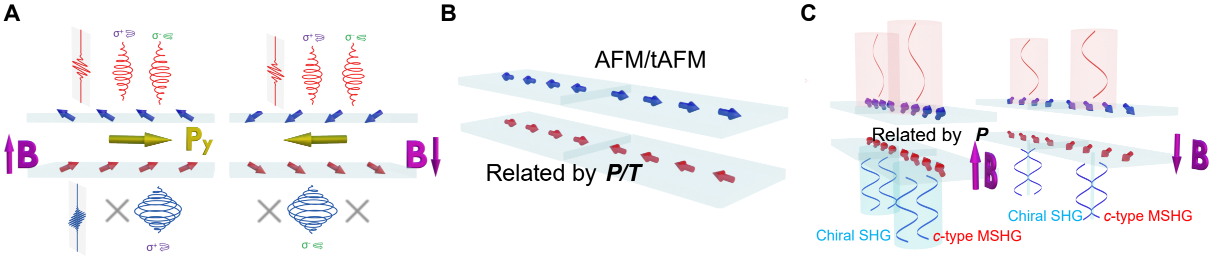

Figure S7: **Schematics of interference effects.** (A) Schematics of LPL/CPL switch effects, CPL filter effects and spin-canting-induced electric polarization reversal under the reversal of **B**. (B) Schematics of the coexistence of AFM structures and its $\mathcal{T}$-reversal pair (tAFM) in BL CrSBr. (C) Schematics of interference effects in different domains under the reversal of **B**.

Fig. S7A depicts the interference effects in BL cAFM CrSBr. For the incident of LPL with the polarization along the yellow line shown in Fig. 4D of the main text, or for the incident of CPL at photon energies around the yellow line in Fig. 4C of the main text, the reversal of **B** can switch on/off the outgoing LPL/CPL SHG, serving as an optical switch. Additionally, for CPL incident at photon energies around the yellow line in Fig. 4C of the main text, the material can selectively emit SHG with specific helicity, resulting in a giant SHG circular dichroism effect, which can serve as an optical filter. It is worth noting that these interference effects could not only greatly modulate the SHG signal but also reveal the reversal of spin-canting-induced electric polarization, enabling them to serve as detectors of magnetoelectric effects.

Figs. S7 (B and C) depict the insensitivity of interference effects in BL CrSBr to the reversal of the AFM domains. Due to energy degeneracy, two types of magnetic domains with opposite Néel vectors may coexist, that is, the AFM and tAFM domains. As $\mathcal{PT}$ symmetry exists in the AFM state, the two domains are related by either $\mathcal{T}$ operation or $\mathcal{P}$ operation, as shown in Fig. S7B. When an external **B** field is applied, while the $\mathcal{T}$ symmetry no longer exists between the two domains, the $\mathcal{P}$ symmetry still connect the two opposite AFM domains. Consequently, both the c-type MSHG and the chiral SHG susceptibilities have opposite signs in opposite domains. However, the global minus sign induced by the $\mathcal{P}$ operation between domains does not manifest in the SHG intensity, as depicted in Fig. S7C. Therefore, the constructive and destructive interference effects are exactly the same between the opposite domains.

1.5 Supplement of SHG under different magnetic states of BL CrSBr

In the main text, the c-type MSHG susceptibility $|\chi^{xxx}|$ and the chiral SHG susceptibility $|\chi^{yyy}|$ are taken as examples to demonstrate the behaviors of SHG under the variation of θ within $(\theta, 0°)$ canting states and the variation of φ within $(10°, \varphi)$ canting states. Under the same process of canting angle variation, the direct proportionality of $\sin 2\theta$ and $\cos\theta$ is also observed in other chiral and c-type MSHG components, as shown in Fig. S8. Similarly, the direct proportionality of $\cos\varphi$ is observed in other chiral SHG and c-type MSHG components, as shown in Fig. S9. These results reveal the robustness of our phenomenological theory.

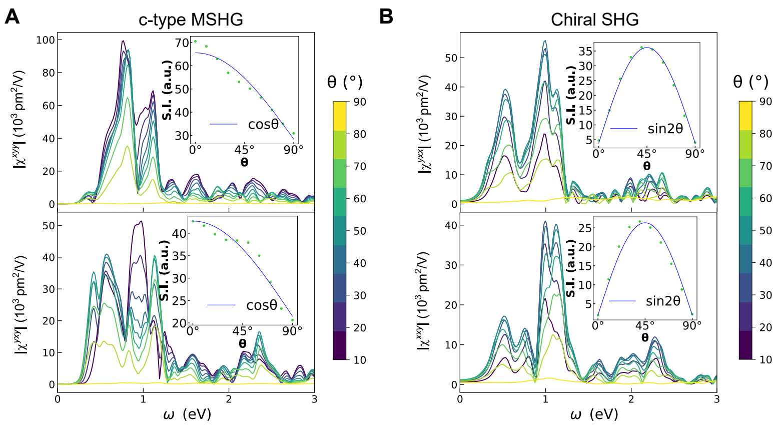

Figure S8: **The dependence of SHG on θ within the canting configuration $(\theta, 0°)$ in BL cAFM CrSBr.** The dependence of the c-type MSHG susceptibilities $|\chi^{xyy}|$, $|\chi^{yxy}|$ (A), and the chiral SHG susceptibilities $|\chi^{yxx}|$, $|\chi^{xxy}|$ (B) on θ is shown. The insets represent spectral integrals of corresponding SHG susceptibilities.

In the main text, the directly proportional relationship between chiral SHG susceptibility $|\chi^{yyy}|$ and spin-canting-induced electric polarization P_y is taken as an example to demonstrate the strong correlations between chiral SHG and P_y under variation of θ within $(\theta, 0°)$ canting

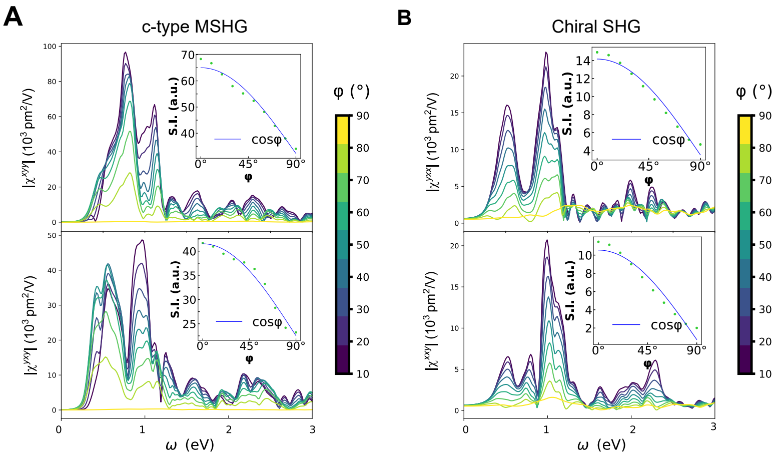

Figure S9: **The dependence of SHG on φ within the canting configuration (10°, φ) in BL cAFM CrSBr.** The dependence of the c-type MSHG susceptibilities $|\chi^{xyy}|$, $|\chi^{yxy}|$ (A), and the chiral SHG susceptibilities $|\chi^{yxx}|$, $|\chi^{xxy}|$ (B) on φ is shown. The insets represent spectral integrals of corresponding SHG susceptibilities.

states and variation of φ within $(10°, \varphi)$ canting states. Under the same process of canting angle variation, this direct proportionality relationship is also observed in other chiral SHG components, as shown in Fig. S10. These results reveal the robustness of their correlation.

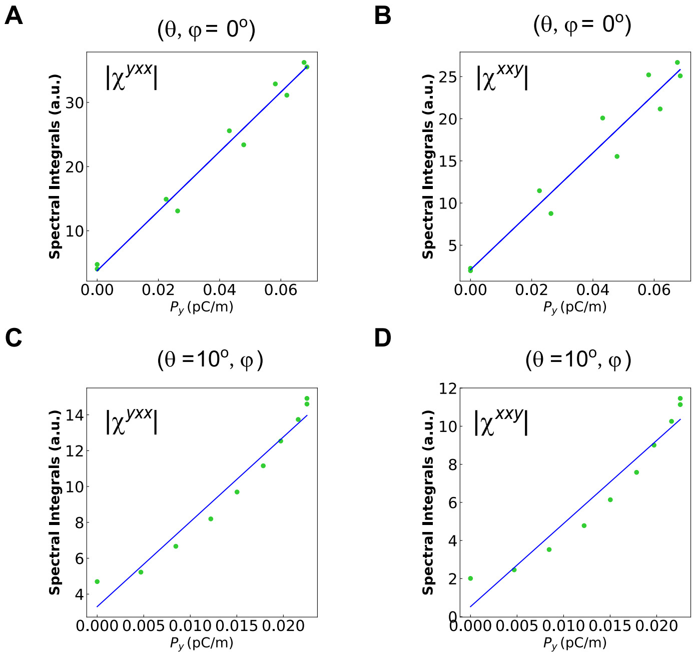

Figure S10: **Magnetoelectric detection in BL cAFM CrSBr.** The relationship between the spectral integrals of the chiral SHG susceptibilities $|\chi^{yxx}|$ (A) and $|\chi^{xxy}|$ (B) with spin-canting-induced electric polarization under the variation of θ within the $(\theta, 0°)$ canting states. The relationship between the spectral integrals of the chiral SHG susceptibilities $|\chi^{yxx}|$ (C) and $|\chi^{xxy}|$ (D) with spin-canting-induced electric polarization under the variation of φ within the $(10°, \varphi)$ canting states.

In addition to the $(\theta, 0°)$ and $(10°, \varphi)$ canting states discussed in the main text, where both c-type MSHG and chiral SHG coexist, the $(0°, \varphi)$ canting states are also worth discussing. $(0°, \varphi)$ canting states belong to the magnetic point group $22'2'$, in which the coexistence of $C_{2y}\mathcal{T}$ and C_{2x} only allows nonzero c-type MSHG susceptibilities χ^{xxx}, χ^{xyy} and χ^{yxy}, as shown in Table 2 of the main text. Figure S11A depicts this spin canting process under the increase of an in-plane magnetic field $\mathbf{B}$ along the $\hat{x}$-axis. The corresponding influence on c-type MSHG susceptibilities χ^{xxx}, χ^{xyy} and χ^{yxy} under this process is shown in Fig. S11B. The spectral integrals of all components are found to be proportional to $\cos\varphi$ and $|\mathbf{L}|$. This consistency with our phenomenological theory in the main text provides a way to tune the c-type MSHG solely with an in-plane $\mathbf{B}$ field.

In addition to the canting states of BL cAFM CrSBr discussed in the main text and above, the rotation of the Néel vector in BL AFM CrSBr is another interesting case deserving of investigation. It should be noted that the $\mathcal{PT}$ symmetry persists in this process, resulting in the exclusive presence of the c-type MSHG. As depicted in Fig. S12A, when the Néel vector rotates within the xy-plane and deviates from the $\hat{y}$-axis, the C_{2x} symmetry is broken, resulting

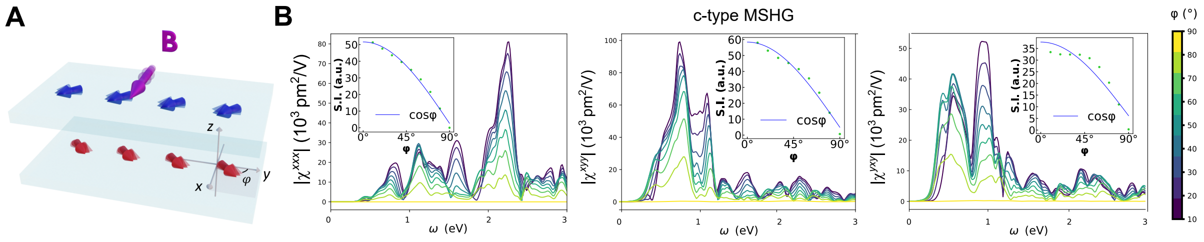

Figure S11: **The dependence of SHG on φ within the canting configuration (0°,φ) in BL cAFM CrSBr.** (A) Schematics of the in-plane canting process with **B** along the $\hat{x}$-axis. (B) The dependence of the c-type MSHG $|\chi^{xxx}|$, $|\chi^{xyy}|$, and $|\chi^{yxy}|$. The insets represent spectral integrals of corresponding SHG susceptibilities.

in the presence of χ^{yyy}, χ^{yxx}, and χ^{xxy}. When the Néel vector is along the $\hat{x}$-axis, the emergent C_{2y} symmetry will forbid the χ^{xxx}, χ^{xyy}, and χ^{yxy} components. Taking $|\chi^{xxx}|$ and $|\chi^{yyy}|$ as examples, $|\chi^{xxx}|$ shows a decay behavior of $\cos\varphi$ while $|\chi^{yyy}|$ shows an increase behavior of $\sin\varphi$ with increasing φ from 0° to 90°, as shown in Fig. S12C. To understand these behaviors, we established a phenomenological theory based on their order parameters, as in the main text. Under rotation of the Néel vector within the xy-plane, χ^{xxx} (χ^{xyy} and χ^{yxy}) requires nonzero $\hat{y}$ components of the Néel vector L_y to break the C_{2y} symmetry, while χ^{yyy} (χ^{xxy} and χ^{xxy}) requires nonzero $\hat{x}$ components of the Néel vector L_x to break the C_{2x} symmetry. Generally, the two sets of SHG should be functions of their order parameters L_y and L_x, and can be expanded in series form

$$\begin{aligned} \chi^{xxx}\,(\chi^{xyy}, \chi^{yxy}) &\propto \sum_i A_i L_y^{2i+1}\,, \\ \chi^{yyy}\,(\chi^{yxx}, \chi^{xxy}) &\propto \sum_i B_i L_x^{2i+1}. \end{aligned} \tag{S14}$$

Here, i and j are nonnegative integers, and A_i (B_i) are coefficients of different terms. If we only keep the leading linear term, the SHG susceptibilities become

$$\begin{aligned} \chi^{xxx}\,(\chi^{xyy}, \chi^{yxy}) &\propto A_0 L_y \propto \cos\varphi\,, \\ \chi^{yyy}\,(\chi^{yxx}, \chi^{xxy}) &\propto B_0 L_x \propto \sin\varphi. \end{aligned} \tag{S15}$$

The expanded phenomenological theory effectively explains the behaviors of SHG, demonstrating the robustness of our theory. Furthermore, it offers a unique approach to detect the rotation of the Néel vector within the xy-plane by monitoring the SHG signal.

Similarly, another typical process of rotation of the Néel vector occurs within the yz-plane, as depicted in Fig. S12B. The upper panel of Fig. S12D shows a decay behavior of $\cos\varphi$ of the $|\chi^{xxx}|$ component, which is very similar to that in Fig. S12C. This can be explained by the aforementioned phenomenological theory, as $\chi^{xxx} \propto A_0 L_y \propto \cos\theta$. However, the persisting C_{2x} symmetry forbids the components χ^{yyy} (χ^{yxx}, χ^{xxy}), as shown in the lower panel of Fig. S12D. This observation highlights the robustness of our theory and also provides a new way to detect the rotation of the Néel vector within the yz-plane by monitoring the SHG signal.

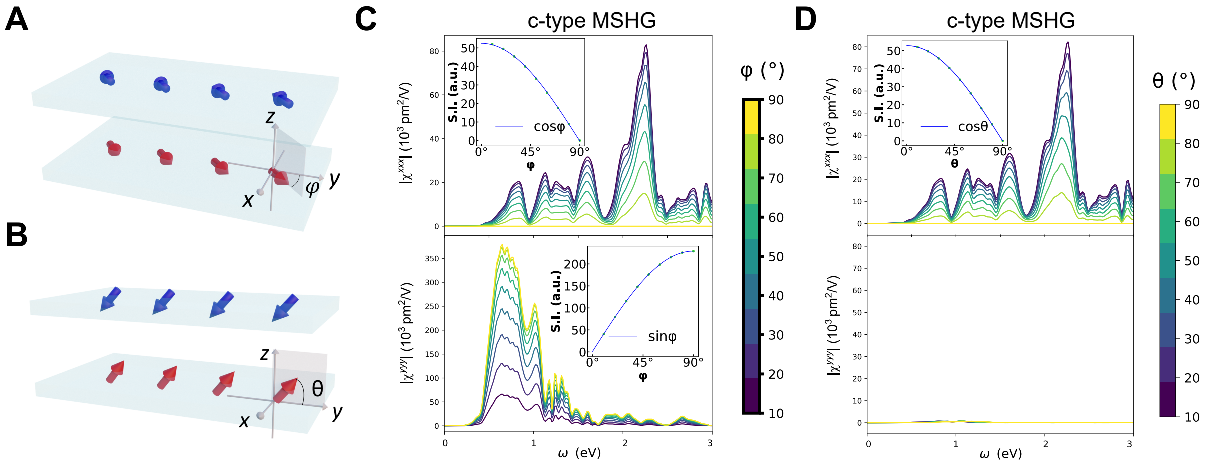

Figure S12: **The dependence of SHG on the rotation of the Néel vector in BL AFM CrSBr.** Schematics of the rotation of the Néel vector within the xy-plane (A), and corresponding influences on the c-type MSHG components $|\chi^{xxx}|$ and $|\chi^{yyy}|$. Schematics of the rotation of the Néel vector within the yz-plane (B), and corresponding influences on the c-type MSHG components $|\chi^{xxx}|$ and $|\chi^{yyy}|$ (C and D).

1.6 SHG diode effects induced by chiral SHG

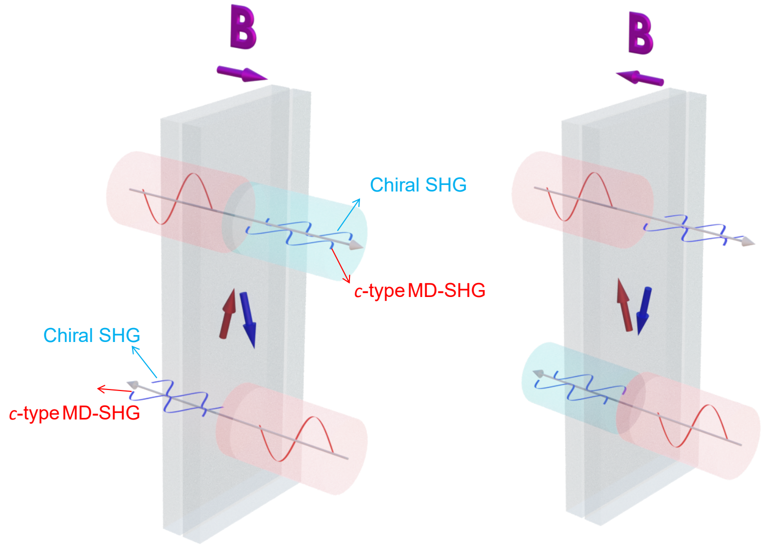

Figure S13: **SHG diode effects.** The schematics of the SHG diode effects induced by chiral SHG and c-type MD-SHG in BL cAFM CrSBr.

The high tunability of chiral SHG under different canting angles enables the appearance of interference effects beyond ED-SHG. A tiny canting-angle-induced chiral SHG can have a magnitude comparable to a higher-order SHG, such as magnetic-dipole SHG (MD-SHG) (*3*). When 2D CrSBr has the $(\theta \neq 0°, 0°)$ canting configuration, c-type MD-SHG emerges with the same tensor components as chiral SHG χ^{yyy}, χ^{yxx} and χ^{xxy}. Under the reversal of the propagation direction of light, represented by the reversal of the light wave vector $\pm\mathbf{k}$, MD-SHG reverses its sign, while the chiral SHG remains unchanged, resulting in interference $I^{\pm\mathbf{k}} = |(\chi_{\text{chiral}} \pm \chi_{\text{MD}})|^2$, which is known as the SHG diode effect (*45*, *46*). The maximal interference due to the comparable chiral SHG and MD-SHG can serve as the SHG diode as shown in Fig. S13, similar to that in Ref. (*45*) but in a more tunable fashion. Again, the $\mathcal{PT}$ operation induced by the reversal of the spin-canting direction reverses the sign of i-type chiral SHG but retains the sign of c-type MD-SHG. As a consequence, it reverses the unidirectional behavior of this SHG diode, as shown in Fig. S13.

2 SHG of other 2D magnets

2.1 SHG of BL cAFM CrX_3 (X=I,Cl)

To validate the generality and transferability of our theoretical predictions for CrSBr, we conducted further investigations on other typical 2D magnets, CrI_3 and $CrCl_3$, which are known to host A-type AFM along the out-of-plane and in-plane directions, respectively. To reveal the SHG responses at spin-canting states, we investigated the cases of CrI_3 under an in-plane magnetic field along the $\hat{x}$-axis and $CrCl_3$ under an out-of-plane magnetic field along the $\hat{z}$-axis.

Both BL AB-stacking CrI_3 and $CrCl_3$ share centrosymmetric crystal structures with the crystal point group S_6. According to the canting angle θ defined for BL CrI_3 in Fig. S14A and for BL $CrCl_3$ in Fig. S15A, the Néel vector and vector spin chirality can be derived as

$$
\begin{aligned}
|\mathbf{L}| &= |\mathbf{S_A} - \mathbf{S_B}| \propto |\cos\theta| \,, \\
|\boldsymbol{\kappa}| &= |\mathbf{S_A} \times \mathbf{S_B}| \propto |\sin 2\theta| \,.
\end{aligned}
\tag{S16}
$$

Therefore, similar to the phenomenological theory described by Eqs. (4-5) in the main text, the spectral integral of the norm of chiral SHG and c-type MSHG is expected to be proportional to $\sin 2\theta$ and $\cos\theta$, respectively. Compared to CrSBr, both CrI_3 and $CrCl_3$ exhibit lower symmetry when subjected to canting, allowing for the simultaneous presence of both chiral SHG and c-type MSHG in each individual SHG component, as summarized in Fig. S14B and Fig. S15B. Thus, we separately calculated i-type and c-type SHG for each single SHG component, using the formulas in Section S3.2.

As shown in Fig. S14(c-d), chiral SHG with a magnitude comparable to c-type MSHG is observed in BL cAFM CrI_3, and the $\sin 2\theta$ and $\cos\theta$ dependencies of chiral SHG and c-type MSHG are clearly observed in all SHG components, highlighting the generality of our phenomenological theory. However, as shown in Fig. S15 (C and D), all MSHG in $CrCl_3$ are almost vanishing, with small residual values insensitive to the canting angle, which can be attributed to numerical noise. We attributed the vanishing of MSHG in $CrCl_3$ to its much larger interlayer spacing (8.76 Å) compared to CrI_3 (6.71 Å), as well as its weaker SOC strength in Cl compared to I. To verify this, we reduced the interlayer spacing to half of its equilibrium value (d_0), that is, 0.5 d_0, as depicted in Fig. S16A, with its symmetry remaining unchanged (Fig. S16B). Remarkably, the much enhanced chiral SHG with a $\sin 2\theta$ dependence and c-type MSHG with a $\cos\theta$ dependence are observed, confirming the dependence of MSHG on interlayer spacing and aligning with the discussions in the main text.

A

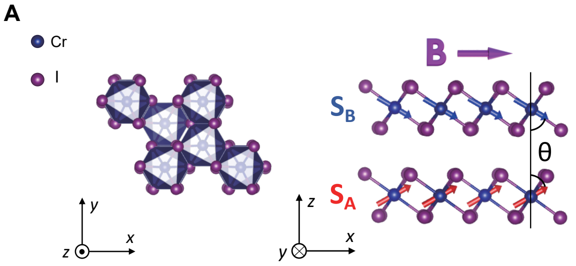

B

AB CrI_3	$\theta = 0°$	$0° < \theta < 90°$	$\theta = 90°$
MPGs	$\bar{3}'$	1	$\bar{1}$
Chiral SHG	N/A	xxx, xyy, yxy<br>yyy, yxx, xxy	N/A
c-type MSHG	$xxx = -xyy = -yxy$<br>$yyy = -yxx = -xxy$	xxx, xyy, yxy<br>yyy, yxx, xxy	N/A

C

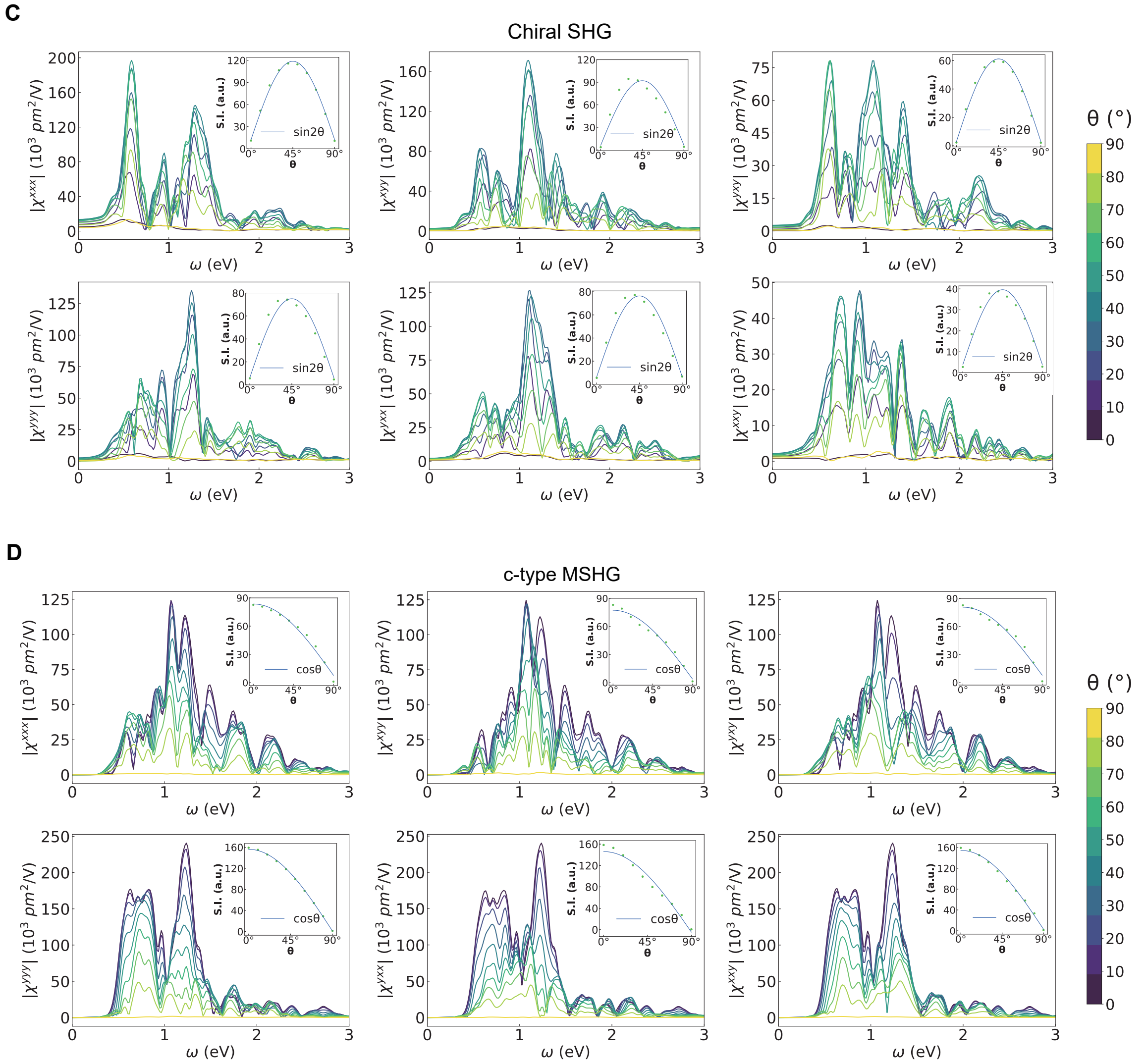

Figure S14: **MSHG in BL AB-stacking CrI_3 under an in-plane magnetic field.** (A) The crystal and magnetic structures of BL CrI_3 under an in-plane magnetic field. (B) Magnetic point groups of BL CrI_3 with different spin canting configurations and their corresponding nonzero SHG components. (C) Chiral SHG and (D) c-type MSHG components for different spin-canting configurations. The insets show the corresponding spectral integrals.

A

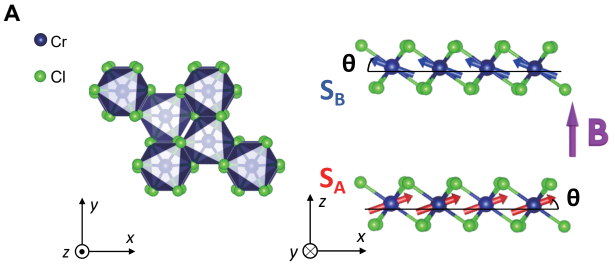

B

AB $CrCl_3$	$\theta = 0°$	$0° < \theta < 90°$	$\theta = 90°$
MPGs	$\bar{1}'$	1	$\bar{3}$
Chiral SHG	N/A	xxx, xyy, yxy yyy, yxx, xxy	N/A
c-type MSHG	xxx, xyy, yxy yyy, yxx, xxy	xxx, xyy, yxy yyy, yxx, xxy	N/A

C

D

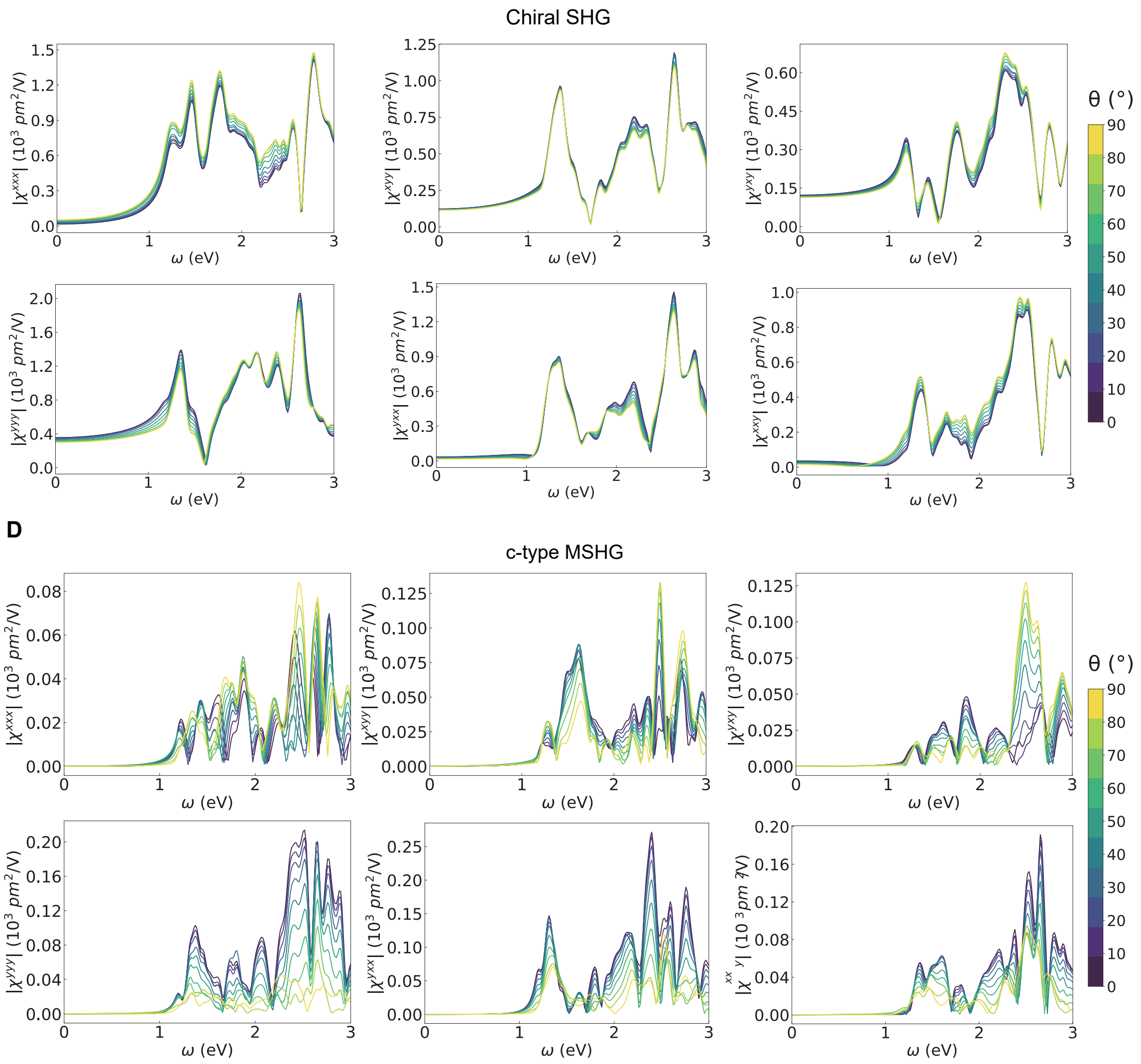

Figure S15: **MSHG in BL AB-stacking $CrCl_3$ under an out-of-plane magnetic field.** (A) The crystal and magnetic structures of BL $CrCl_3$ under an out-of-plane magnetic field. (B) Magnetic point groups of BL $CrCl_3$ with different spin canting configurations and their corresponding nonzero SHG components. (C) Chiral SHG and (D) c-type MSHG components for different spin-canting configurations. The insets show the corresponding spectral integrals.

A

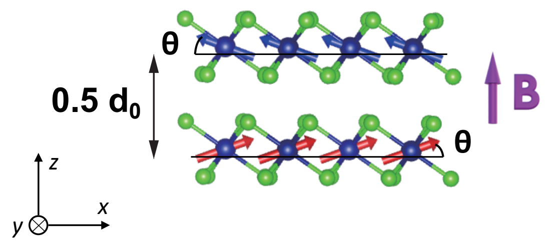

B

AB $CrCl_3$ 0.5 d_0	$\theta = 0°$	$0° < \theta < 90°$	$\theta = 90°$
MPGs	$\bar{1}'$	1	$\bar{3}$
Chiral SHG	N/A	xxx, xyy, yxy yyy, yxx, xxy	N/A
c-type MSHG	xxx, xyy, yxy yyy, yxx, xxy	xxx, xyy, yxy yyy, yxx, xxy	N/A

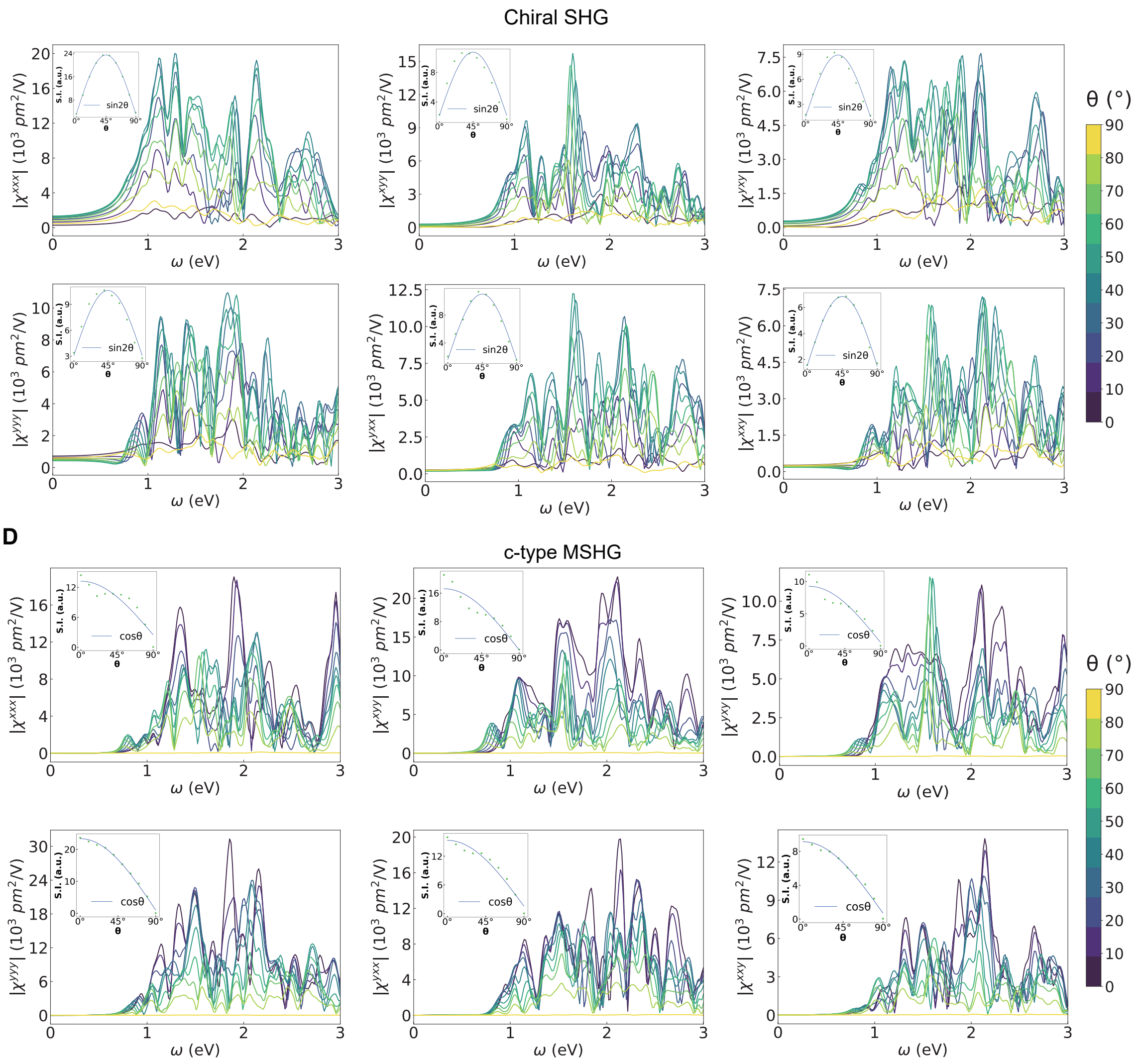

Figure S16: **MSHG in BL AB-stacking $CrCl_3$ with reduced interlayer spacing under an out-of-plane magnetic field.** (A) The schematic of BL $CrCl_3$ with reduced interlayer spacing under an out-of-plane magnetic field. (B) Magnetic point groups of BL $CrCl_3$ with different spin canting configurations and their corresponding nonzero SHG components. (C) Chiral SHG and (D) c-type MSHG components for different spin-canting configurations. The insets show the corresponding spectral integrals.

2.2 Interference effects of BL cAFM CrX_3 (X=I,Cl)

For both CrI_3 and $CrCl_3$ with a reduced spacing between layers, notable interference effects in SHG are observed. However, due to the lower symmetry in these cases, the interference effects are more complex compared to CrSBr, as shown in Fig. S17 and Fig. S18.

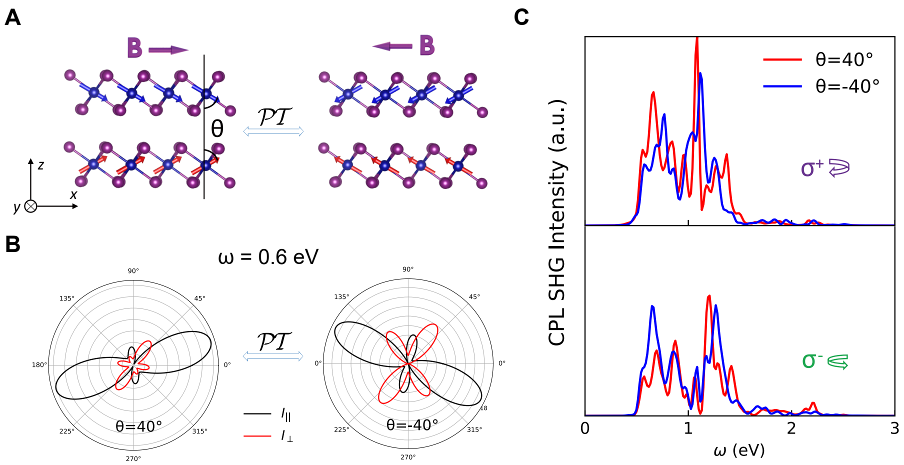

Figure S17: **Interference effects of chiral SHG in BL cAFM CrI_3.** (A) Schematics of the reversal of spin canting direction in BL cAFM CrI_3 with reduced interlayer spacing. (B) The polarization-resolved SHG at $\omega = 0.6$ eV. (C) Circularly polarized SHG intensity of $\mathcal{PT}$-related canting states for $\theta = \pm 40°$.

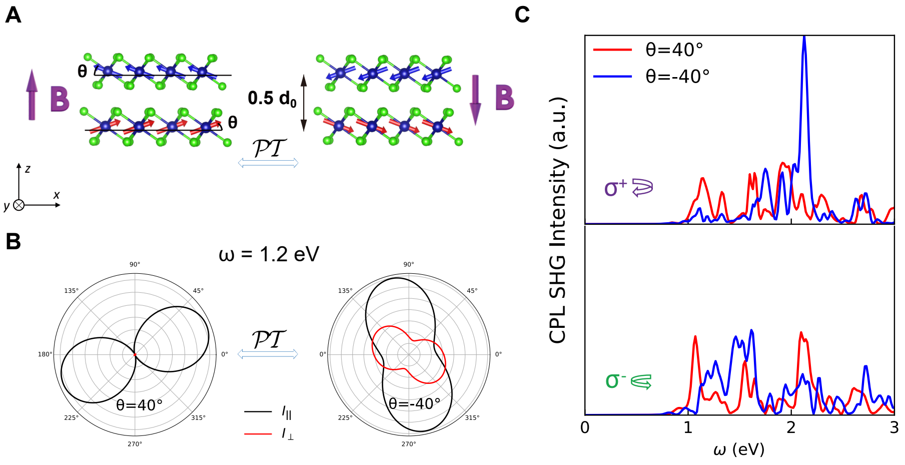

Figure S18: **Interference effects of chiral SHG in BL cAFM $CrCl_3$ with reduced interlayer spacing.** (A) Schematics of the reversal of spin canting direction in BL cAFM $CrCl_3$ with reduced interlayer spacing. (B) The polarization-resolved SHG at $\omega = 1.2$ eV. (C) Circularly polarized SHG intensity of $\mathcal{PT}$-related canting states for $\theta = \pm 40°$.

3 General theory of optical responses

3.1 Symmetry requirements of SHG

Electric-dipole SHG susceptibility, χ^{abc}, is a third-rank tensor with the $\mathcal{P}$-odd property, $\mathcal{P}\chi^{abc} = -\chi^{abc}$, and a permutation symmetry, $\chi^{abc} = \chi^{acb}$. It can generally be divided into $\mathcal{T}$-even (i-type) terms $\chi^{abc}_{(i)}$ and $\mathcal{T}$-odd (c-type) terms $\chi^{abc}_{(c)}$. The transformations of them under a symmetry operation O can be expressed as follows (*4*)

$$\chi^{abc}_{(i)/(c)} = (+/-)O_{al}O_{bm}O_{cn}\chi^{lmn}_{(i)/(c)} \, . \tag{S17}$$

Here, O_{ab} denotes the elements of the coordinate transformation matrix. When the $\mathcal{T}$-operation is involved in symmetry operation O, the i-type tensors yield the '+' sign while the c-type tensors yield the '−' sign in Eq. S17. For magnetic materials without $\mathcal{P}$ but with $\mathcal{PT}$ symmetry, $\chi^{abc}_{(i)}$ is forbidden while $\chi^{abc}_{(c)}$ is allowed (*4*)

$$\begin{cases} \mathcal{PT}\chi^{abc}_{(i)} = -\chi^{abc}_{(i)} = \chi^{abc}_{(i)} \Rightarrow \chi^{abc}_{(i)} = 0 \, , \\ \mathcal{PT}\chi^{abc}_{(c)} = \chi^{abc}_{(c)} \, , \end{cases} \tag{S18}$$

For non-magnetic materials without $\mathcal{P}$ but with $\mathcal{T}$ symmetry, $\chi^{abc}_{(c)}$ is forbidden while $\chi^{abc}_{(i)}$ is allowed (*4*)

$$\begin{cases} \mathcal{T}\chi^{abc}_{(i)} = \chi^{abc}_{(i)} \, , \\ \mathcal{T}\chi^{abc}_{(c)} = -\chi^{abc}_{(c)} = \chi^{abc}_{(c)} \Rightarrow \chi^{abc}_{(c)} = 0 \, , \end{cases} \tag{S19}$$

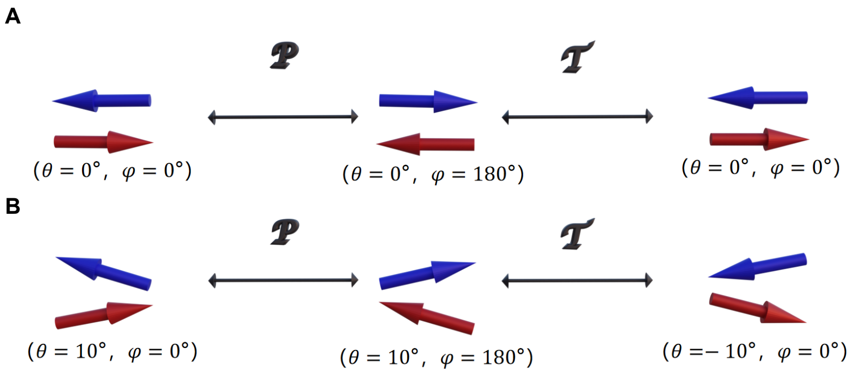

Figure S19: **The symmetry breaking process by magnetic ordering.** The influence of $\mathcal{P}$ and $\mathcal{T}$ operations on the AFM system (A) and cAFM system (B). The definitions of θ and φ are consistent with those in Fig. 2B of the main text. The red and blue arrows represent magnetic moments in different magnetic sublattices.

Now we are in a position to show that the i-type SHG with a magnetic origin can be achieved by breaking $\mathcal{PT}$ symmetry via noncollinear magnetic ordering. As shown in Fig. S19A, under

$\mathcal{P}$ operation, the collinear AFM system cannot return to itself, but it can return to itself after $\mathcal{PT}$ operation, demonstrating the absence of $\mathcal{P}$ symmetry but presence of $\mathcal{PT}$ symmetry in this system. In contrast, when spins are canted, the noncollinear cAFM system cannot return to itself under $\mathcal{P}$ or $\mathcal{PT}$ operations, demonstrating the simultaneous breaking of $\mathcal{P}$ and $\mathcal{PT}$ symmetries, as shown in Fig. S19B. Therefore, the *i*-type SHG with a magnetic origin can be activated in this cAFM system.

3.2 Microscopic formula of SHG

The SHG susceptibility tensors were calculated following our previous work (*28*, *29*). The interband contribution to SHG for the *i*-type term is

$$\begin{aligned}&\chi^{abc}_{e,(i)}(-2\omega;\omega,\omega)\\&=\frac{e^3}{2\hbar^2}\int[d\mathbf{k}]\sum_{l,m,n}\frac{\mathrm{Re}[r^a_{nm}(r^b_{ml}r^c_{ln}+r^c_{ml}r^b_{ln})]}{\omega_{ln}-\omega_{ml}}\left(\frac{2f_{nm}}{\omega_{mn}-2\omega}+\frac{f_{ln}}{\omega_{ln}-\omega}+\frac{f_{ml}}{\omega_{ml}-\omega}\right).\end{aligned}\tag{S20}$$

The interband contribution to SHG for the *c*-type term is

$$\begin{aligned}&\chi^{abc}_{e,(c)}(-2\omega;\omega,\omega)\\&=\frac{ie^3}{2\hbar^2}\int[d\mathbf{k}]\sum_{l,m,n}\frac{\mathrm{Im}[r^a_{nm}(r^b_{ml}r^c_{ln}+r^c_{ml}r^b_{ln})]}{\omega_{ln}-\omega_{ml}}\left(\frac{2f_{nm}}{\omega_{mn}-2\omega}+\frac{f_{ln}}{\omega_{ln}-\omega}+\frac{f_{ml}}{\omega_{ml}-\omega}\right).\end{aligned}\tag{S21}$$

The SHG expression contributed from both interband and intraband processes for the *i*-type term is

$$\begin{aligned}&\chi^{abc}_{i,(i)}(-2\omega;\omega,\omega)\\&=\frac{-e^3}{2\hbar^2}\int[d\mathbf{k}]\sum_{m,n}f_{nm}\left[\frac{2\mathrm{Im}[r^a_{nm}(r^b_{mn;c}+r^c_{mn;b})]}{\omega_{mn}(\omega_{mn}-2\omega)}+\frac{\mathrm{Im}[r^b_{mn}r^a_{nm;c}+r^c_{mn}r^a_{nm;b}]}{\omega_{mn}(\omega_{mn}-\omega)}\right.\\&\left.+\frac{\mathrm{Im}[r^a_{nm}(r^b_{mn}\Delta^c_{mn}+r^c_{mn}\Delta^b_{mn})]}{\omega^2_{mn}}\left(\frac{1}{\omega_{mn}-\omega}-\frac{4}{\omega_{mn}-2\omega}\right)-\frac{\mathrm{Im}[r^c_{mn}r^b_{nm;a}+r^b_{mn}r^c_{nm;a}]}{2\omega_{mn}(\omega_{mn}-\omega)}\right].\end{aligned}\tag{S22}$$

The SHG expression contributed from both interband and intraband processes for the c-type term is

$$\begin{aligned}
&\chi^{abc}_{i,(c)}(-2\omega;\omega,\omega)\\
=&\frac{ie^3}{2\hbar^2}\int[d\mathbf{k}]\sum_{m,n} f_{nm}\left[\frac{2\mathrm{Re}[r^a_{nm}(r^b_{mn;c}+r^c_{mn;b})]}{\omega_{mn}(\omega_{mn}-2\omega)}+\frac{\mathrm{Re}[r^b_{mn}r^a_{nm;c}+r^c_{mn}r^a_{nm;b}]}{\omega_{mn}(\omega_{mn}-\omega)}\right.\\
&+\frac{\mathrm{Re}[r^a_{nm}(r^b_{mn}\Delta^c_{mn}+r^c_{mn}\Delta^b_{mn})]}{\omega^2_{mn}}\left(\frac{1}{\omega_{mn}-\omega}-\frac{4}{\omega_{mn}-2\omega}\right)\\
&\left.-\frac{\mathrm{Re}[r^c_{mn}r^b_{nm;a}+r^b_{mn}r^c_{nm;a}]}{2\omega_{mn}(\omega_{mn}-\omega)}+\frac{\mathrm{Re}[\Delta^a_{mn}(r^b_{nm}r^c_{mn}+r^c_{nm}r^b_{mn})]}{4\omega^2_{mn}(\omega_{mn}-\omega)}\right].
\end{aligned}\tag{S23}$$

In the above expressions, the small imaginary part $i\eta$ in the denominator, which also acts as the broadening factor of the Dirac delta function, is not written explicitly. Here, ω can be regarded as $\omega + i\eta$, where $\eta = \hbar/\tau$ and τ is the quantum decoherence time (see Section S1.3). The value $\eta = 0.05$ eV, which corresponds to $\tau = 13$ fs, is adopted in the SHG calculations.

3.3 Microscopic formula of the linear optical absorption spectrum

The expression for the dielectric function is

$$\epsilon^{ab} = \delta_{ab} + \frac{e^2}{\hbar}\int\frac{d^3\boldsymbol{k}}{(2\pi)^3}\sum_{n,m}\frac{f_{nm}r^a_{nm}r^b_{mn}}{\omega_{mn}-\omega-i\eta}. \tag{S24}$$

where η in the denominator is the broadening factor of the Dirac delta function. The imaginary part of ϵ represents the linear optical absorption spectrum. The joint density of states (JDOS) is defined as

$$\int\frac{d^3\boldsymbol{k}}{(2\pi)^3}\sum_{n,m} f_{mn}\delta(\omega_{mn}-\omega) \tag{S25}$$
